# Prescriptions of Prof. Linton, of St. Louis, for Dropsy

**Published:** 1847-02

**Authors:** 


*Prescriptions of Prof. Linton, of St. Louis, for Dropsy.*—

| | | |
|---|---|---|
| ℞. Sup. Tart. Potas. | - - - | 2 dr. |
| Jalap. | - - - - - | ½ dr. |
| Gamboge, | - - | gr. 1 M. |

The preparations of iron, are we believe, the best tonics which can be used in those cases of dropsy, (and they are numerous) in which a roborant treatment is indicated.

We cannot help regarding a sort of old woman's prescription, a roughly prepared acetate of iron made by putting a handful of rusty nails into a pint of old cider, as one of the best, if not *the best*, preparation for this invaluable tonic. We have "seen with our eyes" its happy effects in scores of cases, to say nothing of that less to be credited testimony—what we have "heard with our ears."—*St. Louis Med. and Surg. Jour.*

